# Supplementary material for: Modification of Xanthan Gum for a High-Temperature and High-Salinity Reservoir
Source: Polymers (Basel). 2021 Dec 1;13(23):4212. doi: 10.3390/polym13234212 (PMC8659541; doi:10.3390/polym13234212)
Supplement: Supplementary file 1 [file polymers-13-04212-s001.zip › polymers-1373152-supplementary.pdf]

Supporting Information

Modification of Xanthan Gum for a High Temperature and Salinity Reservoir

Mohamed Said[1], Bashirul Haq[1, *], Dhafer Al Shehri[1], Mohammad Mizanur Rahman[2], Nasiru Salahu Muhammed[1] and Mohamed Mahmoud[1].

[1] College of Petroleum Engineering and Geosciences, King Fahd University of Petroleum & Minerals, Dhahran, 34464, Saudi Arabia

[2] Interdisciplinary Research Center for Advanced Materials, King Fahd University of Petroleum & Minerals, Dhahran, 34464, Saudi Arabia

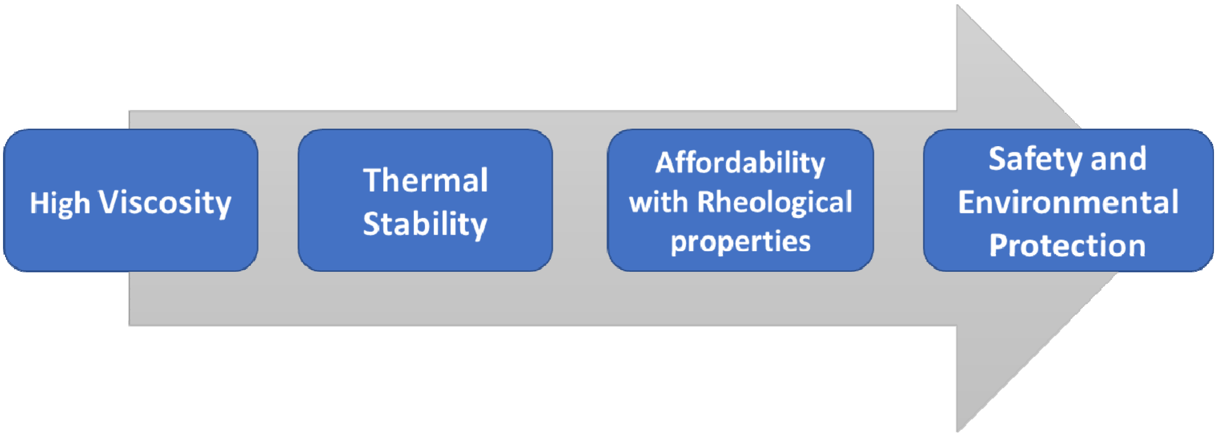

Figure S1. Advantages of xanthan gum (Gbadamosi et al., 2019; Muhammed et al., 2020).

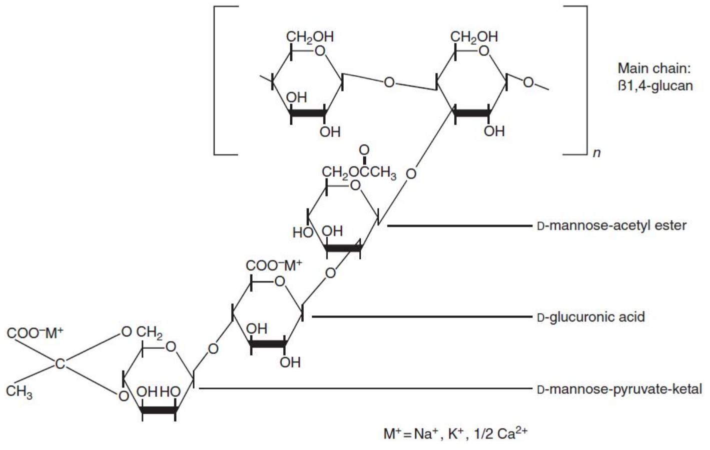

Figure S2. Chemical structure of xanthan gum (Wever et al., 2011).

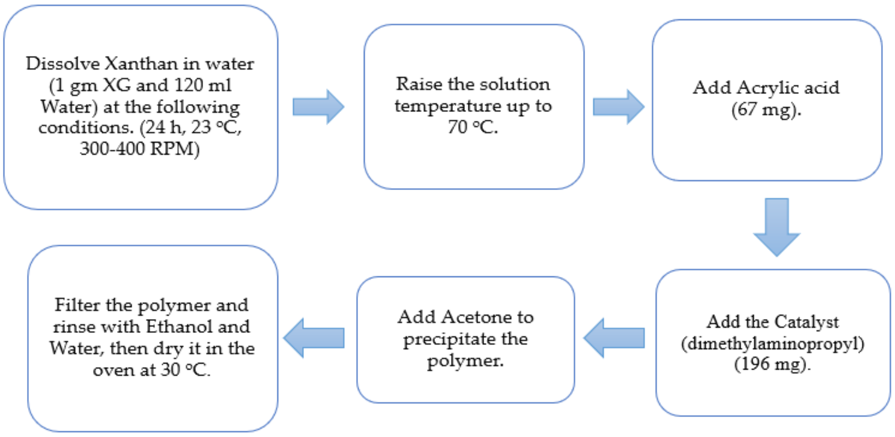

Figure S3. Process flow diagram of chemical synthesis.

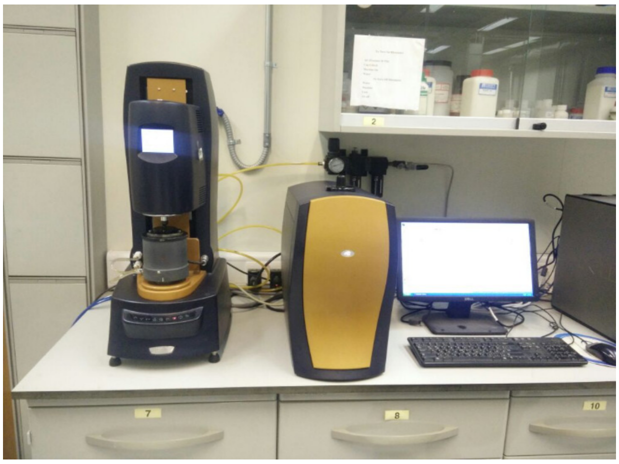

Figure S4. The Discovery hybrid rheometer.

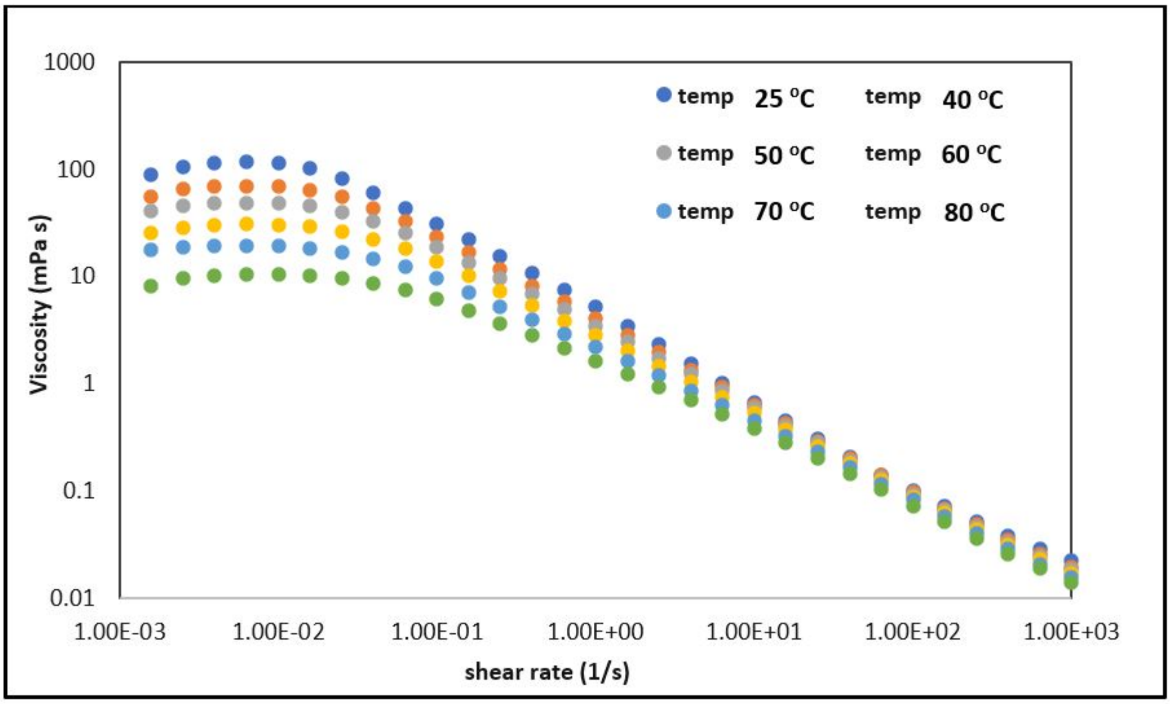

Figure S5. 0.5% xanthan viscosity vs. shear rate at different temperatures.

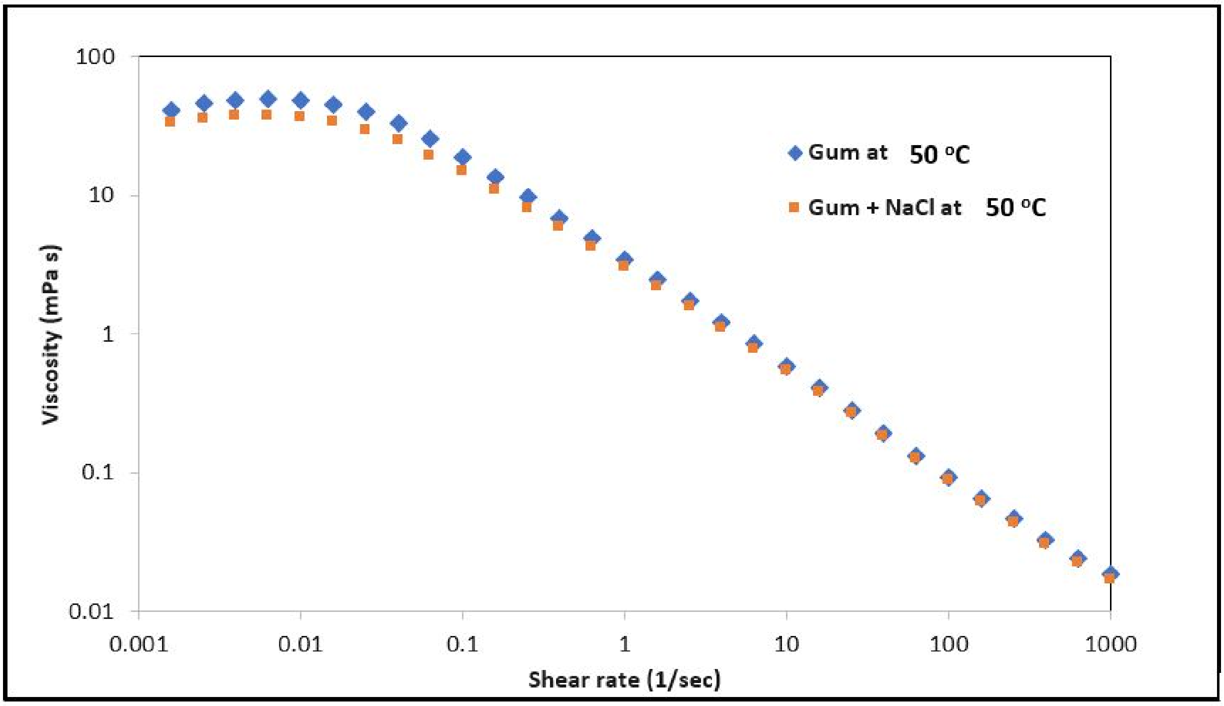

Figure S6. Effect of adding NaCl to 0.5% xanthan viscosity vs. shear rate at 50 °C.

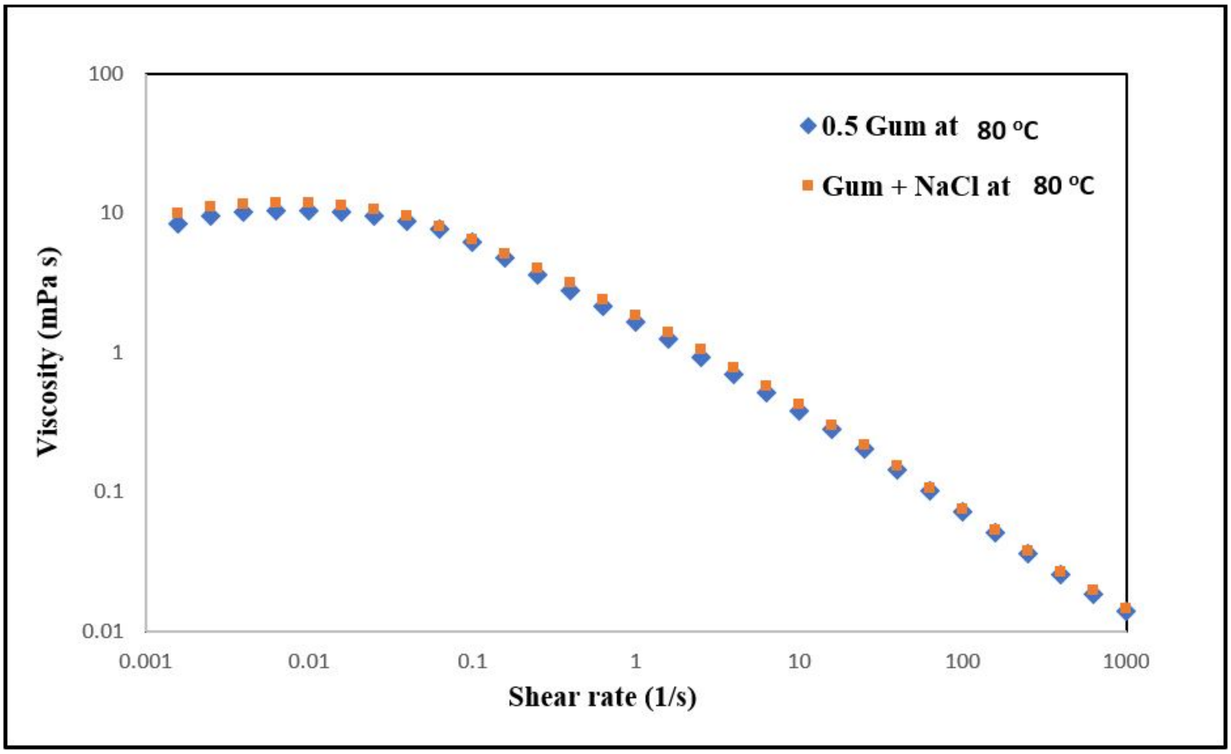

Figure S7. Effect of adding NaCl to 0.5% xanthan viscosity vs. shear rate at 80 °C.

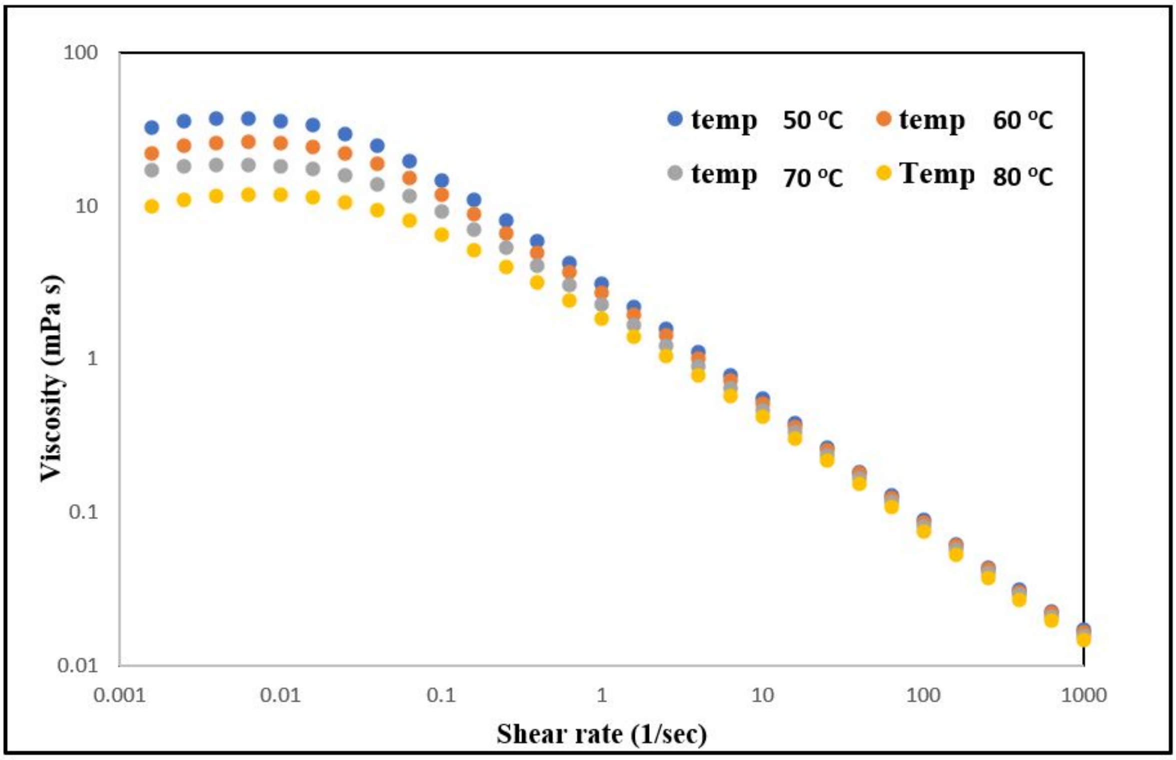

Figure S8. 0.5% xanthan (3% NaCl) vs. shear rate at different temperatures.

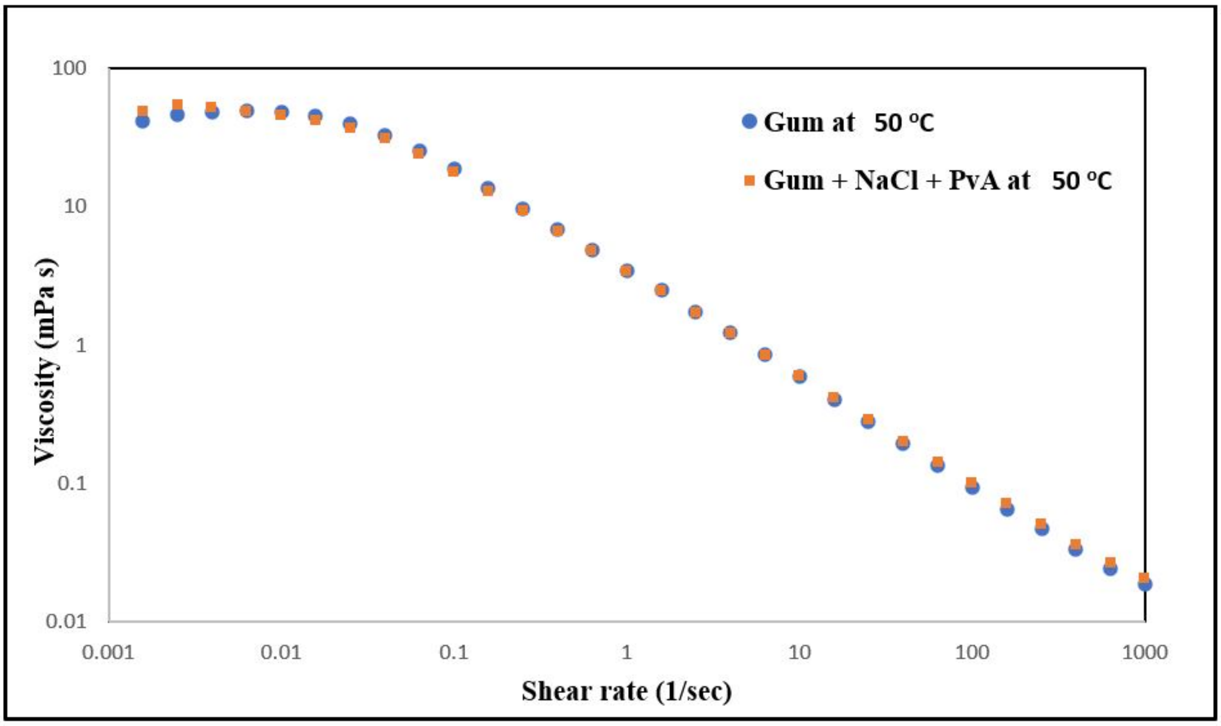

Figure S9. Effect of adding PVA to 0.5% xanthan viscosity vs. shear rate at 50 ºC.

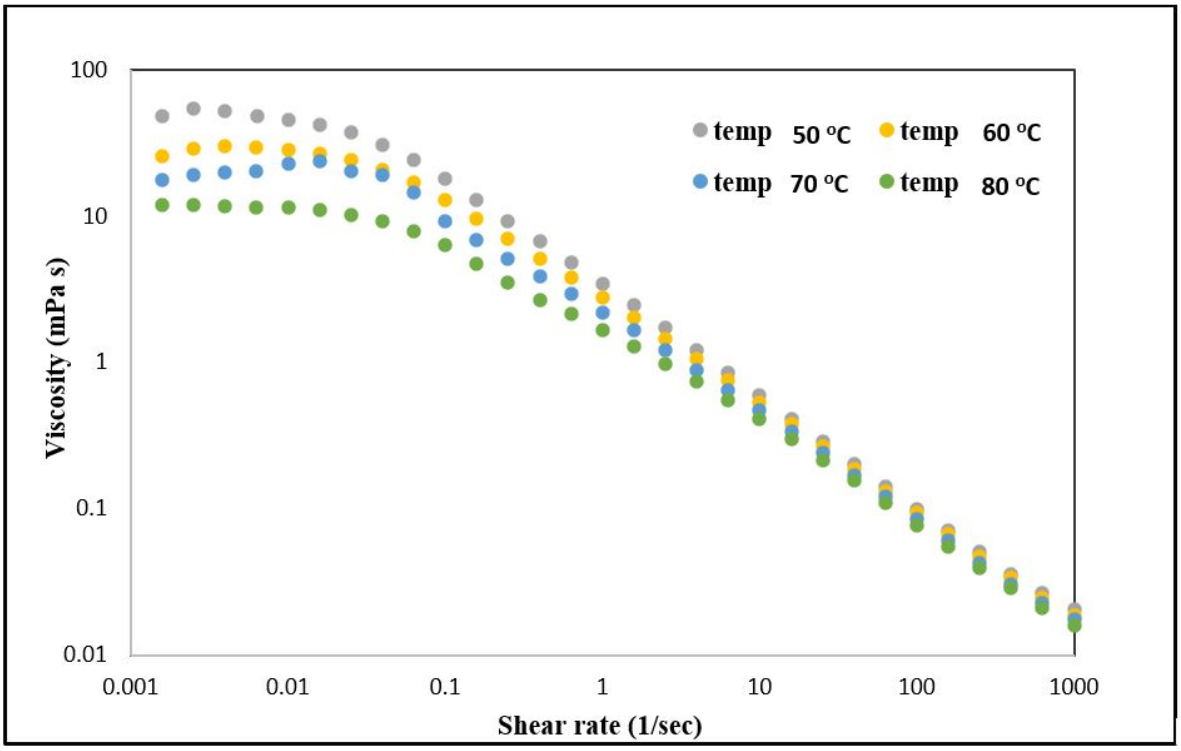

Figure S10. 0.5% xanthan viscosity vs. shear rate + 0.5% PVA at different temperatures.

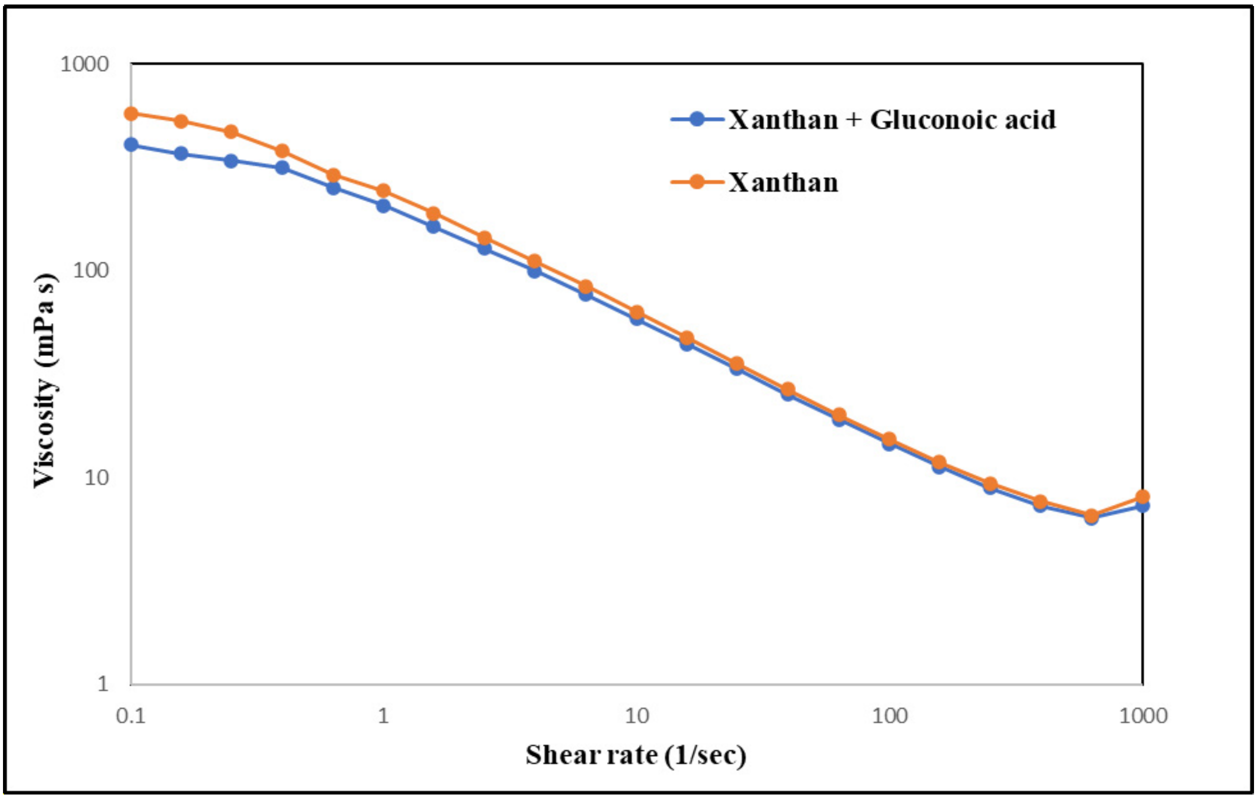

Figure S11. 1500 ppm xanthan and xanthan + gluconic acid viscosities vs. shear rate at 25 °C.

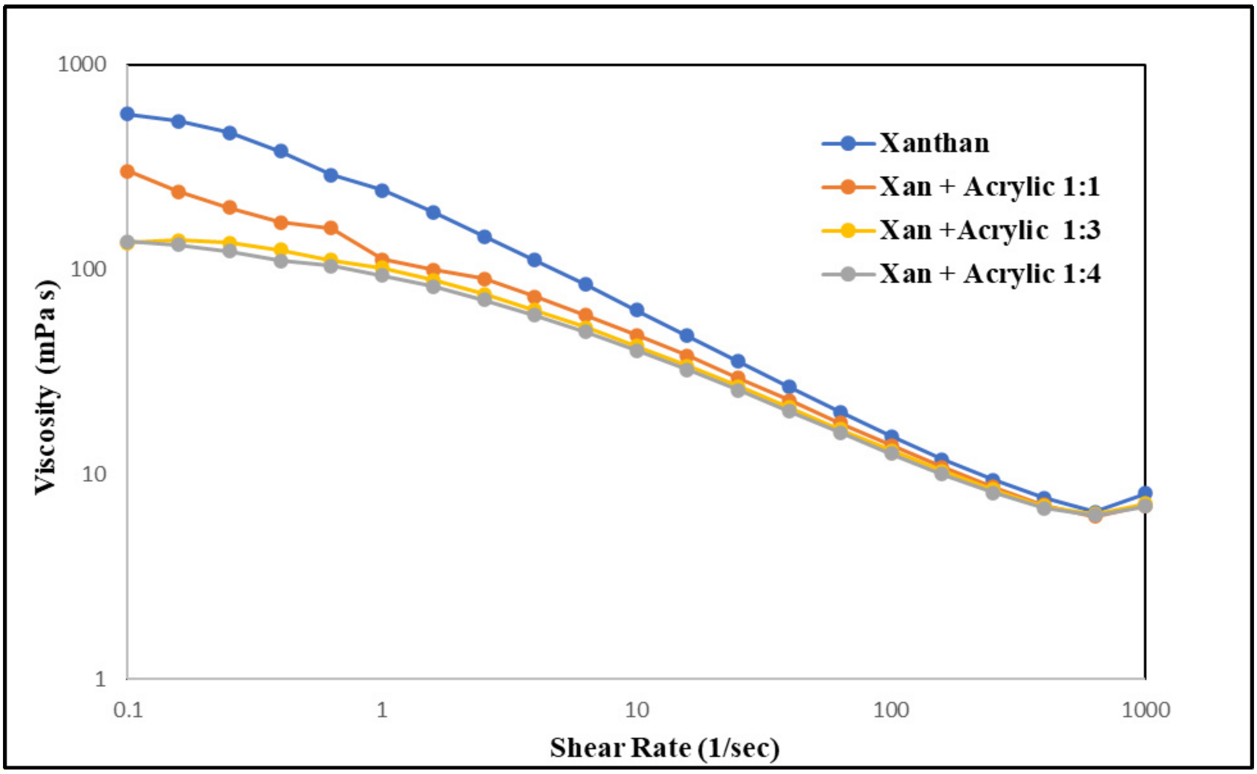

Figure S12. 1500 ppm xanthan and xanthan + acrylic acid (blending) viscosities vs. shear rate at 50 °C.

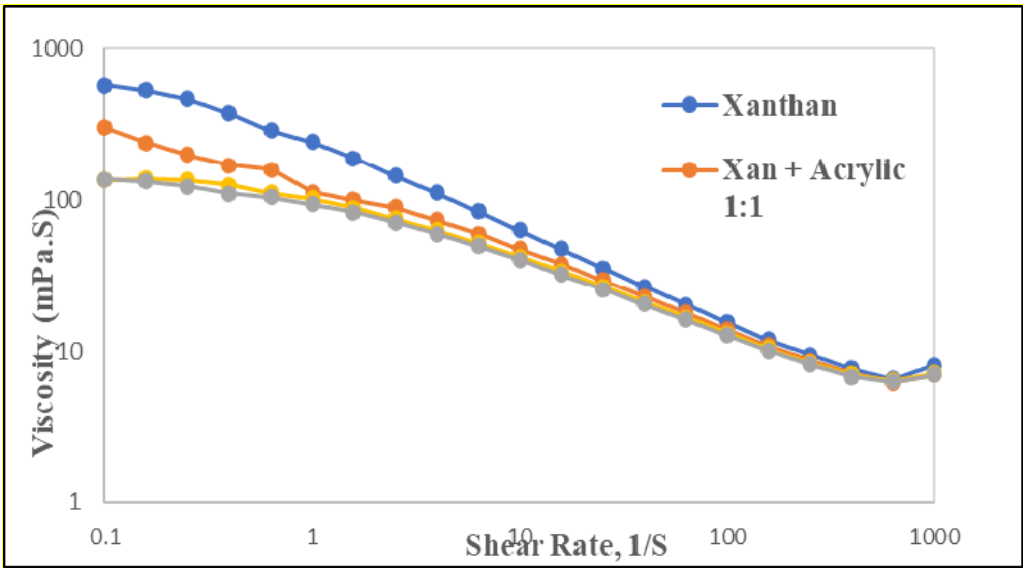

Figure S13. 1500 ppm xanthan and xanthan + acrylic acid (blending) viscosities vs. shear rate at 25 °C.

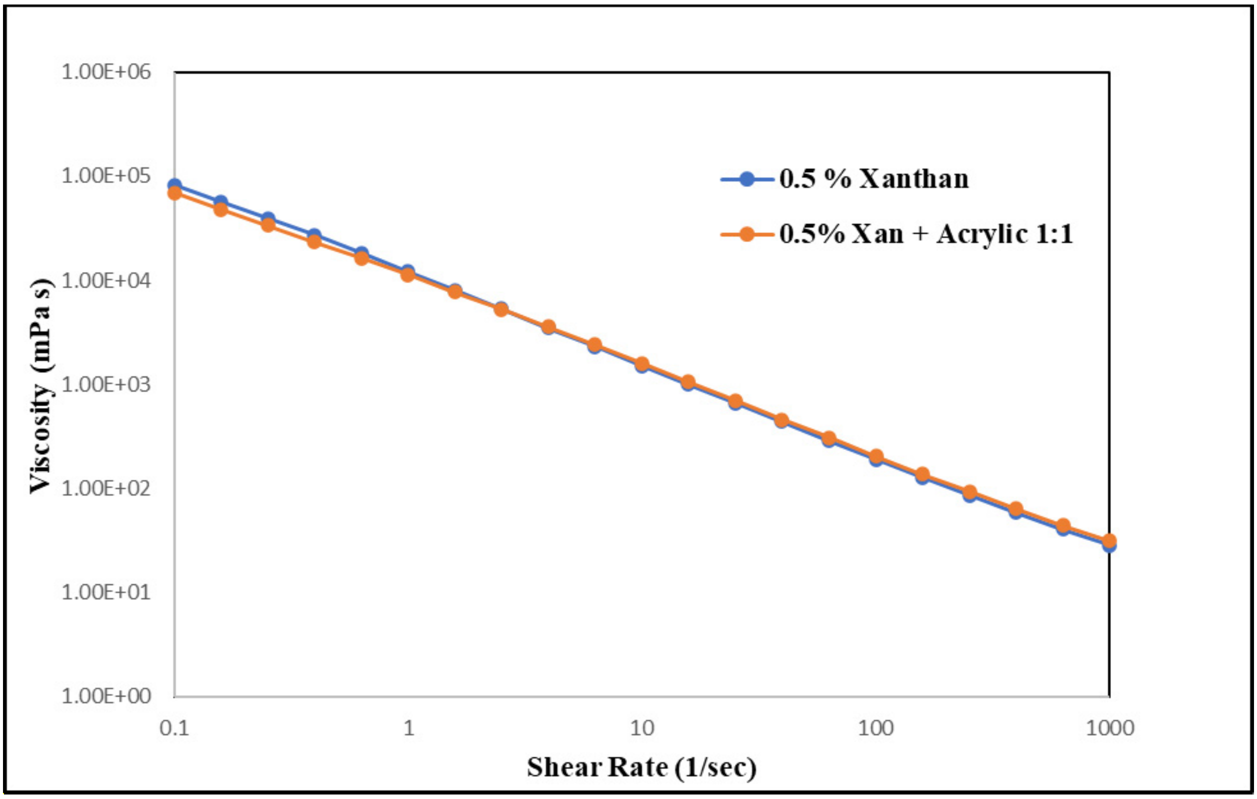

Figure S14. 0.5% xanthan and xanthan + acrylic acid (blending) viscosities vs. shear rate at 50 °C.

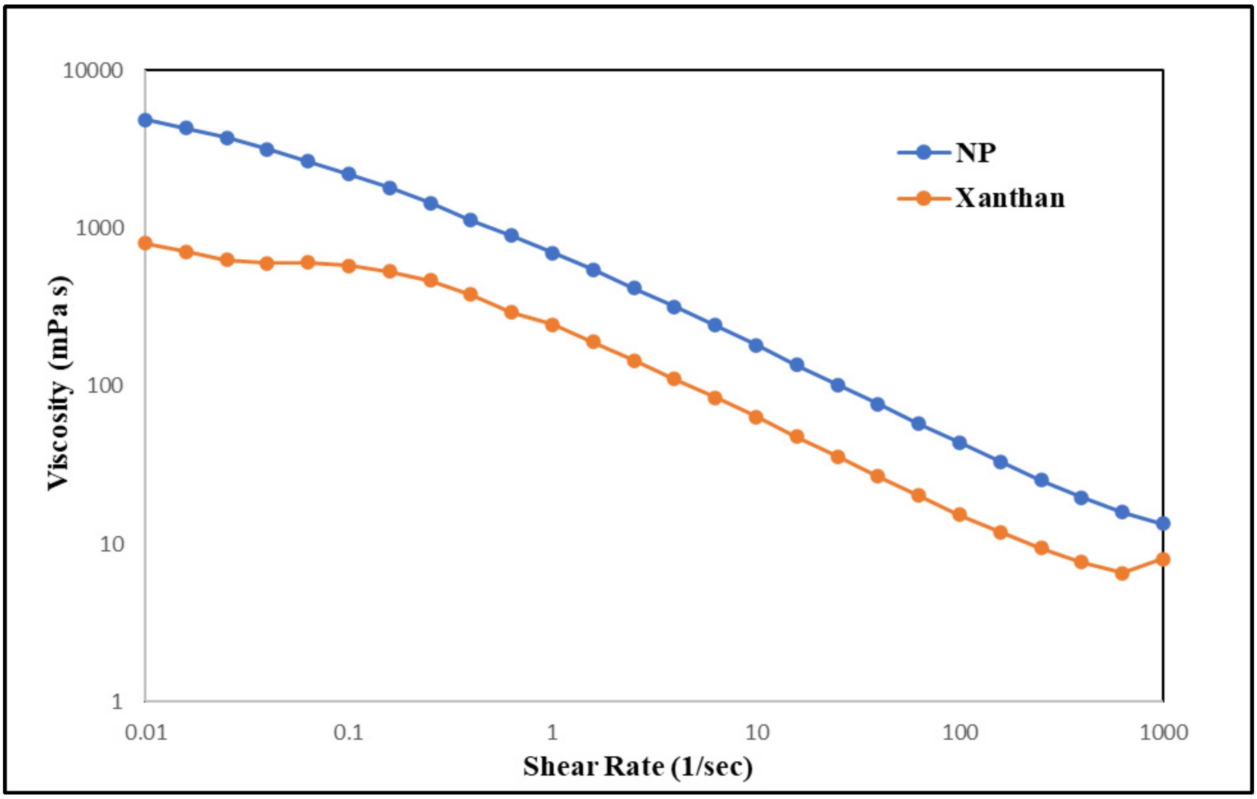

Figure S15. Xanthan Acrylate and xanthan viscosities vs. shear rate at 25 °C.

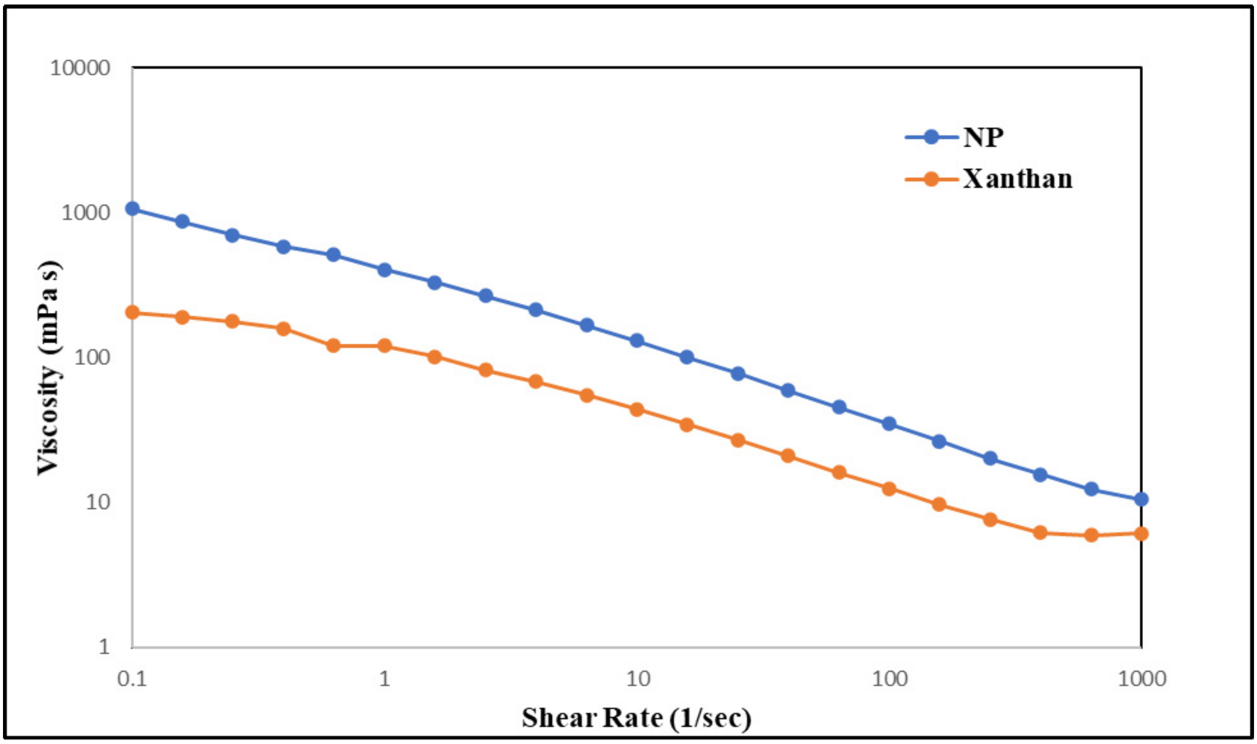

Figure S16. Xanthan Acrylate and xanthan viscosities vs. shear rate at 50 °C.

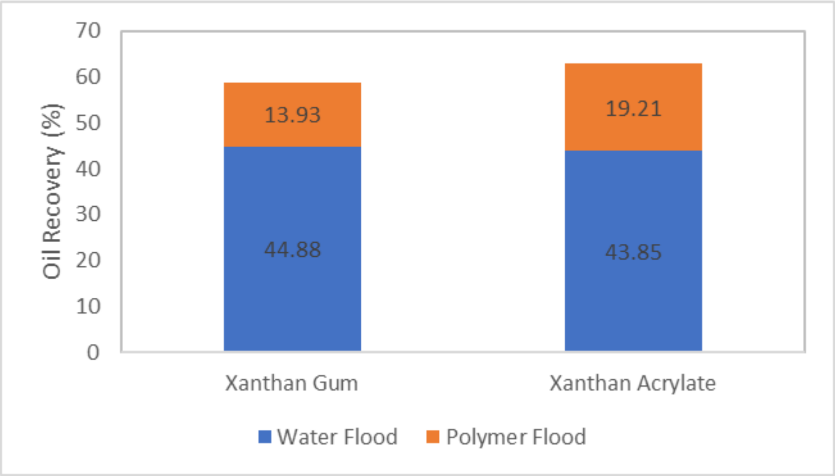

Figure S17. Comparison of oil recovery between xanthan gum and the new polymer.
